# Is having quality as an item on the executive board agenda associated with the implementation of quality management systems in European hospitals: a quantitative analysis

**DOI:** 10.1093/intqhc/mzu017

**Published:** 2014-02-17

**Authors:** Daan Botje, N.S. Klazinga, R. Suñol, O. Groene, H. Pfaff, R. Mannion, A. Depaigne-Loth, O.A. Arah, M. Dersarkissian, C. Wagner, on behalf of the DUQuE Project Consortium, N. Klazinga, D.S. Kringos, M.J.M.H. Lombarts, T. Plochg, M.A. Lopez, P. Vallejo, F. Saillour-Glenisson, M. Car, S. Jones, E. Klaus, S. Bottaro, P. Garel, M. Saluvan, C. Bruneau, A. Depaigne-Loth, A. Hammer, O. Ommen, H. Pfaff, D. Botje, A. Escoval, A. Lívio, M. Eiras, M. Franca, I. Leite, F. Almeman, H. Kus, K. Ozturk, R. Mannion, A. Wang, A. Thompson

**Affiliations:** 1 NIVEL, Netherlands Institute for Health Services Research, Utrecht, The Netherlands; 2 Department of Public Health, Academic Medical Center, University of Amsterdam, Amsterdam, The Netherlands; 3 Avedis Donabedian Research Institute (FAD), Universitat Autonoma de Barcelona, Spain; 4 Red de investigación en servicios de salud en enfermedades crónicas REDISSEC, Spain; 5 Department of Health Services Research and Policy, London School of Hygiene and Tropical Medicine, London, UK; 6 Institute of Medical Sociology, Health Services Research and Rehabilitation Science, University of Cologne, Cologne, Germany; 7 Health Services Management Centre, University of Birmingham, Birmingham B15 2RT, UK; 8 Haute Autorité de la Sante, Paris, France; 9 Department of Epidemiology, Fielding School of Public Health, University of California, Los Angeles (UCLA), Los Angeles, CA, USA; 10 UCLA Center for Health Policy Research, Los Angeles, CA, USA; 11 Department of Public and Occupational Health, EMGO Institute for Health and Care Research, VU University Medical Center, Amsterdam, The Netherlands

**Keywords:** quality management, executive board, quality on the agenda, external pressure, international research, acute care hospitals

## Abstract

**Objective:**

To assess whether there is a relationship between having quality as an item on the board's agenda, perceived external pressure (PEP) and the implementation of quality management in European hospitals.

**Design:**

A quantitative, mixed method, cross-sectional study in seven European countries in 2011 surveying CEOs and quality managers and data from onsite audits.

**Participants:**

One hundred and fifty-five CEOs and 155 quality managers.

**Setting:**

One hundred and fifty-five randomly selected acute care hospitals in seven European countries (Czech Republic, France, Germany, Poland, Portugal, Spain and Turkey).

**Main outcome measure(s):**

Three constructs reflecting quality management based on questionnaire and audit data: (i) Quality Management System Index, (ii) Quality Management Compliance Index and (iii) Clinical Quality Implementation Index. The main predictor was whether quality performance was on the executive board's agenda.

**Results:**

Discussing quality performance at executive board meetings more often was associated with a higher quality management system score (regression coefficient *b* = 2.53; SE = 1.16; *P* = 0.030). We found a trend in the associations of discussing quality performance with quality compliance and clinical quality implementation. PEP did not modify these relationships.

**Conclusions:**

Having quality as an item on the executive board's agenda allows them to review and discuss quality performance more often in order to improve their hospital's quality management. Generally, and as this study found, having quality on the executive board's agenda matters.

## Introduction

Executive boards in hospitals are under increasing pressure to assure and improve the quality of care delivered to patients. They have to improve management systems to meet the demands of a wide range of stakeholders [1]. External pressures such as accreditation [2, 3], publicly available performance data [4, 5] and market competition [6] have been associated with organizational changes. Since executive boards have the responsibility of daily management of the hospital, external pressures can play a pivotal role in the prioritization and agenda-setting of the executive board. Agenda setting is considered to be the starting point of prioritization and decision-making [7], and having quality on the agenda should allow executive board members to review and discuss the quality and performance of the services they deliver to patients.

However, it is not self-evident that executive boards drive the quality and safety agenda within their organizations. Executive boards tend to primarily focus on the hospital's financial health, at the expense of considering quality and safety issues [8–10]. The lack of active engagement of executive boards in quality of care aligns with the growing concern that (financial) incentives within healthcare systems are often failing to support quality improvement (QI), i.e. the business case for quality is lacking [11].

In previous studies, the executive board's engagement was found to be associated with the successful implementation of QI projects [12–14], for example, by facilitating a supportive IT systems [15] and stimulating clinical involvement in total quality management [16]. Executive board's engagement was also found to be associated with adequately funded and systematically evaluated quality management systems [17, 18]. A quality management system has been defined as a set of interacting activities, methods and procedures used to monitor, control and improve the quality of care [19], and is considered to be a prerequisite for the successful implementation of single QI projects [20] and to achieve sustainability.

There are different ways to assess quality management systems [21], but whichever assessment was used, the implementation of quality management systems appeared to differ between hospitals [20, 22–25]. Thus far, it remains unclear why the implementation of quality management systems differs.

Against this background, the aims of our study were to (i) determine how often executive boards have quality performance as an item on the agenda, (ii) investigate the relationship between having quality on the board agenda and the implementation of quality management in hospitals, and finally to (iii) explore the influence of external pressure on this relationship.

## Methods

### Participants

This study was part of the ‘Deepening our Understanding of Quality improvement in Europe (DUQuE)’ project, funded by the EU 7th Research Framework Program [26]. Its aims and methods are described elsewhere [27]. The study used a multi-method, cross-sectional design to collect quality-related information from European hospitals between May 2011 and February 2012 [27]. Seven countries were included based on a mix of health and hospital system financing and organization criteria in different geographical areas in Europe. The participating countries comprised the Czech Republic, France, Germany, Poland, Portugal, Spain and Turkey. In each country, 30 hospitals were randomly recruited, subject to them having more than 130 beds and that they treat acute myocardial infarct, hip fracture, stroke and deliveries patients [27].

### Measuring the constructs

We sent a questionnaire to the Chief Executive Officers (CEOs) to assess how frequently quality performance was an item on the Executive board's agenda: (i) never, or during, (ii) a few, (iii) most, or (iv) every meeting.

The implementation of hospital-wide quality management was measured using three constructs that focused on (i) organization-wide systems, (ii) compliance and (iii) clinical quality efforts. The implementation of the quality management system was determined by the construct *Quality Management System Index* (QMSI) [28]. This was based on the quality manager questionnaire and consists of (i) quality policy documents, (ii) quality monitoring by the board, (iii) training of professionals, (iv) formal protocols for infection control, (v) formal protocols for medication and patient handling, (vi) analysing performance of care processes, (vii) analysing performance of professionals, (viii) analysing feedback patient experiences and (ix) evaluating results. The QMSI ranges from 0 to 27. The level of compliance was measured using the construct *Quality Management Compliance Index* (QMCI) [29]. It is based on audit data and assesses (i) quality planning, (ii) monitoring patient/professional opinion, (iii) monitoring quality systems and (iv) improving quality by staff development. The QMCI ranges from 0 to 16. Third, the *Clinical Quality Implementation Index* (CQII) [29], which is also based on audit data, assesses (i) preventing hospital infection, (ii) medication management, (iii) preventing patient falls, (iv) preventing patient ulcers, (v) routine testing of elective surgery patients, (vi) safe surgery practices and (vii) preventing deterioration. The CQII ranges from 0 to 14.

### Perceived external pressure

Perceived external pressure (PEP) reflects the CEO's perception of the influence external factors from outside the hospital have on the hospital's quality management system. Since there was no validated scale to measure PEP, we asked experts from the participating countries to provide an overview of pressures applicable to hospitals in their country. We identified 18 different external influencing factors (Table 2). Because the meaning of these factors might differ across national cultures and regulatory environments, the items were discussed with the national coordinators from each country as part of the translation process. In the questionnaires, CEOs could indicate how much they considered each single item to have influence on their quality management system (0, no influence; 1, moderate influence; 2, major influence). The composite measure of PEP was constructed by the sum score of 18 external influencing factors. Items were not weighted since it was beyond the scope of this study to determine which factors are more important than others. The composite measure for PEP ranges from 0 to 36.

### Data collection process

Country coordinators approached and recruited hospitals for this study. Hospital coordinators forwarded passwords to the CEO and quality manager to access the web-based questionnaires. In total, 188 hospitals agreed to participate. Questionnaires were completed by 177 (RR = 94%) CEOs and 188 quality managers (RR = 100%). We collected audit data during onsite visits within a subsample of 12 hospitals that were randomly selected from the 30 participating hospitals per country.

### Statistical analyses

Data cleaning was performed prior to the statistical analyses. In our data, 53 out of 188 (28.19%) hospitals were missing at least one value for QMSI and 84 out of 188 (44.68%) of hospitals were missing at least one of the external influencing factors that were used to build the PEP score. In order to mitigate this, we used multiple imputation to obtain data sets that were complete on subscale variables used to build QMSI and PEP. The estimated values were based on the items that were completed by the respondent and on the same item answered by other respondents. Instead of a single fixed value, multiple imputation replaces missing values with a set of plausible values to represent practical uncertainty [30]. It generated five complete data sets and these were used to construct scores of QMSI, QMCI, CQII and PEP for hospitals that missed less than half of the variables used to build each scale. Hospitals missing more than half of the variables were excluded from the analyses. This allowed us to increase our final sample size to 155 hospitals. Since only two CEOs indicated that they never discussed quality performance, we combined this answering category with ‘during few meetings’ in further analyses. Audit data (i.e. QMCI and CQII) were collected in 63 of the 155 hospitals.

Descriptive statistics were calculated to describe hospital characteristics (teaching status, ownership and size) and demographic characteristics of participating CEOs and quality managers (gender, age and number of years in job). Descriptive statistics are also reported for the QMSI, QMCI and CQII (outcomes) and how often was quality on the Board's agenda (predictor) in the analysis.

**Figure 1:**
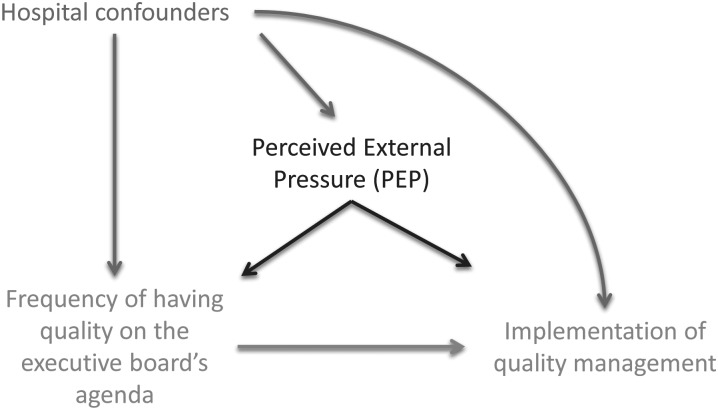
The DAG shows the relationship between the frequency of having quality on the executive board's agenda and the implementation of quality management. Hospital confounders are teaching status, ownership, the number of beds and the country the hospital is situated in. PEP is the CEO's perception of external pressure on the hospital's quality management system. PEP is hypothesized to by a modifier of the relationship between the quality agenda and quality management.

We used the Directed Acyclic Graph (DAG) shown in Fig. 1 to guide our analysis. We analysed the relationship between the frequency of having quality on the executive board's agenda (predictor) with the implementation of quality management as measured by QMSI, QMCI and CQII (outcomes). We used linear random intercept models to estimate associations between each of our three outcomes (QMSI, QMCI, CQII) and the frequency of quality on the agenda and to assess whether PEP modified this predictor–outcome relationship. Models included a random intercept for country in order to account for clustering of hospitals within countries. We also adjusted for hospital confounders such as CEO background in healthcare, hospital teaching status, ownership type and number of beds because we expect that these variables might influence the frequency of having quality on the agenda. To determine statistical differences, the level of significance was set at 5%. All statistical analyses were carried out in SAS (version 9.3, SAS Institute Inc., NC, USA, 2001).

## Results

### Quality on the executive board's agenda

As can be seen in Table 1, the 155 hospitals are non-teaching (55.5%) and teaching (44.5%), and 80% of the hospitals are publicly owned. The executive boards comprised eight members on average. Further analyses showed that the average number of executive board members in France and Spain is more than 10, while in Germany, Poland and Portugal, boards have four or five members (not in the table). CEOs indicated that the executive boards have 36 official meetings per year on average, and 54% had quality performance on the agenda during most meetings. Seventy-nine per cent of the executive boards had at least one member with medical background.

**Table 1:** Descriptive statistics for the outcome, predictors, and characteristics of hospitals, CEOs and quality managers used in the analysis (*n* = 155)^a^

Hospital characteristics		
Teaching status, *n* (%)		
* *Non-teaching	86 (55.48)	
* *Teaching	69 (44.52)	
Ownership, *n* (%)		
* *Private	31 (20.00)	
* *Public	124 (80.00)	
Number of beds, *n* (%)		
* *≤200	15 (9.68)	
* *201–500	66 (42.58)	
* *501–1000	48 (30.97)	
* *>1000	26 (16.77)	
Board size, mean (SD)^b^	7.93 (6.28)	
Members with medical background, *n* (%)		
* *0 members	22 (14.19)	
* *1 member	55 (35.48)	
* *2 members	42 (27.10)	
* *3 or more members	26 (16.77)	
* *Missing	10 (6.45)	
Number of official Board meetings per year, mean (SD)^c^	36.34 (24.80)	
Frequency of quality performance on the agenda of Board meetings, *n* (%)		
* *Never on the agenda for meetings	2 (1.29)	
* *Few meetings	43 (27.74)	
* *Most meetings	84 (54.19)	
* *Every meeting	26 (16.77)	
Perceived external pressure (range 0–36), mean (SD)	19.49 (5.88)	
Quality Management System Index (range 0–27), mean (SD)	19.16 (4.48)	
Quality Management Compliance Index^a^ (range 0–16), mean (SD)	10.52 (3.18)	
Clinical Quality Implementation Index^a^ (range 0–14), mean (SD)	8.39 (2.99)	
Respondent characteristics		
Sex, *n* (%)	CEO	QM
* *Male	132 (85.16)	48 (30.97)
* *Female	23 (14.84)	107 (69.03)
Age (years), mean (SD)	52.64 (6.80)	45.01 (8.81)
Number of years as in job, mean (SD)	5.03 (4.33)	4.61 (3.16)
Background in healthcare		
* *No	14 (9.03)	–
* *Yes	141 (90.97)	–

^a^Audit data were collected in 63 of 155 hospitals in this study. Hospital characteristics of these 63 hospitals were similar to those of the 155 hospitals.

^b^Information on number of executive board members missing in 11 hospitals.

^c^Information on number of executive board meetings in the last year missing in 10 hospitals.

### Quality management

The implementation of quality management was described by QMSI, QMCI and CQII. As shown in Table 1, the average score on QMSI was 19.16 (±4.48) on a scale of 0–27. This means that the majority of hospitals fully implemented quality activities in at least one unit. The QMCI score was 10.52 (±3.18) on a scale of 0–16, meaning that the majority of hospitals demonstrated compliance to a set of activities to plan, control and monitor quality of care. Hospitals scored 8.39 ± 2.99 on CQII (on a scale of 0 to 14), meaning that protocols existed in most of the seven areas of clinical quality efforts, but that they were less frequently monitored and measured.

### Perceptions of external pressure

On the whole, CEOs indicated that their hospital's quality management system is only moderately influenced by external pressures (PEP score = 19.49 ± 5.88 on a scale of 0–36), with an inter-quartile range of 8.6 between the first and the third quartile. ‘Governmental policy’ and ‘legislation for internal quality systems’ were perceived to have the most influence (Table 2), which were similar for all seven countries. Albeit that the external factors that appeared to have the least influence were missing more frequently, the missing values were not considered systematic.

**Table 2:** Descriptive statistics for items used to calculate the PEP score (*n* = 155)

External influencing factors^a^	Mean	SD	Floor (% with lowest score)	Ceiling (% with highest score)	Frequency of missing data
Governmental policy on quality and safety in healthcare	1.59	0.57	4	63	1
Legislation for internal quality systems	1.56	0.58	5	59	4
Public health, sanitary inspection	1.46	0.64	8	53	3
Hospital accreditation	1.45	0.77	16	59	7
Quality system certification (ISO 9004)	1.37	0.76	16	51	8
Statutory inspection to maintain institutional license, registration	1.33	0.73	15	46	5
Accreditation of clinical training	1.29	0.68	12	40	7
Publication disclosure of hospital performance data (e.g. public hospital comparisons, star ratings or league tables)	1.23	0.61	9	32	6
Public relations, media pressure	1.12	0.63	14	25	8
Market competition advantage	1.03	0.68	20	23	10
Requirements for public liability, malpractice insurance	0.90	0.72	24	23	13
Clinical professional associations, colleges, societies	0.87	0.63	25	13	11
Professional chambers/regulators	0.85	0.69	30	16	13
Health insurance funds	0.78	0.76	37	18	16
Technology assessment bodies, e.g. HAS, NICE, SIGN	0.71	0.78	43	17	21
Condition for access to funding, e.g. as ‘preferred provider’	0.66	0.76	45	15	19
Hospital associations	0.57	0.65	47	8	14
Ombudsman	0.57	0.64	46	7	17

^a^All external influencing factors were rated on a scale range of 0–2; 0, no influence; 1, moderate influence; and 2, major influence.

### Quality as an item on the executive board's agenda and the implementation of quality management

Table 3 details the regression coefficients, standard errors and *P*-values from the multivariable adjusted regression models of frequency of having quality performance on the executive board's agenda as predictor of *QMSI, QMCI and CQII*. After adjusting for CEO background in healthcare and hospital characteristics (teaching status, ownership type and size), hospitals where the executive boards had quality on the agenda during every meeting scored 2.532 units higher on QMSI compared with hospitals with executive boards that discussed it during few meetings or never (SE = 1.16; *P* = 0.03). We also looked at modification effect of PEP on the relationship between the frequency of having quality on the agenda and QMSI by redoing the analysis without PEP, but did not find a significant effect.

**Table 3:** Regression coefficients (standard errors) for the associations of having quality performance on the executive board's agenda and hospital level quality measures QMSI, QMCI and CQII, and the modifier PEP

	QMSI^a^ (*n* = 155)		QMCI^a^ (*n* = 63)		CQII^a^ (*n* = 63)	
	*b* (SE)	*P*-value	*b* (SE)	*P*-value	*b* (SE)	*P*-value
Frequency of quality on executive board's agenda						
Every meeting	2.53 (1.16)	0.030	1.23 (1.32)	0.355	1.85 (1.16)	0.117
Most meetings	1.62 (0.78)	0.040	0.86 (0.90)	0.340	1.81 (0.77)	0.023
Never/few meetings	Ref.	Ref.	Ref.	Ref.	Ref.	Ref.
Perceived external pressures score (PEP)	0.06 (0.06)	0.364	0.03 (0.08)	0.701	0.07 (0.07)	0.369

^a^Adjusted for country, CEO background in healthcare, hospital teaching status, ownership type, number of beds. Interaction effects of the quality agenda and PEP were not significant for any outcome measure and therefore not shown.

Table 3 also details the regression coefficients for the frequency of having quality performance on the executive board's agenda as predictor of *QMCI*. Although our results were non-significant, we observed positive associations between frequency of quality on the agenda and QMCI. No effect or modification effect was found for PEP on the association between frequency of quality on the agenda and QMCI.

All else held constant, on average CQII score was higher when executive boards had quality performance on the agenda during most meetings (*b* = 1.81, SE = 0.77, *P* = 0.02) or during every meeting (*b* = 1.85, SE = 1.16, *P* = 0.11), when compared with hospitals where quality was discussed never or at a few meetings. We did not find a modification effect of PEP on this relationship.

## Discussion

The aim of this study was to assess how frequently executive boards in European hospitals have quality as an item on their agenda, and whether this frequency is associated with the implementation of hospital quality management. We have demonstrated that executive boards which take an interest in quality performance, as indicated by having the item on the agenda, are more likely to have a quality management system in place.

Agenda-setting is an important aspect of prioritization [7], and ultimately for hospital governance. In order to take action, executive boards should receive information about quality performance, review and discuss it during meetings, and make the right decision accordingly [31]. Having quality as an item on the executive board's agenda is also important symbolically as it signals their quality orientation to the rest of the hospital, and ultimately obtain more resources than those who do not [32]. Therefore, having quality as an item on the agenda fits well within QI cycles. Frequent discussions of quality performance demonstrate that executive boards consider quality of care to be an important topic and provides the opportunity to ascertain the implementation of the quality management system. The association with quality management could also mean that having a widely implemented quality management system requires executive boards to have many meetings to cover all its different subdimensions. Either way, discussing quality performance seems to be an essential lever for implementing and sustaining hospital-wide quality management. The non-significant trend with compliance (QMCI) and clinical implementation (CQII) of quality management could be attributed to the lower number of hospitals participating in the audit study. However, in order to get a better understanding of the role of executive boards and clinicians in quality management, further and more sustained research is needed to investigate the relationship with quality strategies at pathway level.

Also this article reports on the CEO's ranking of PEP. Although we did not find a modification effect of PEP, we did find that CEOs experienced pressure from external factors. Where external pressure from patient demands, financial pressure and market competition increase, executive boards are under increasing scrutiny to shift their focus in decision-making [33–35]. For example, publicly reported performance data can lead to increased managerial commitment to quality of care, reshape organizational priorities, and create a sense of accountability [5]. In previous studies, quality management systems were found to be more apparent in hospitals that were subject to accreditation and certification [36, 37].

### Limitations

Common limitations of the DUQuE project are described elsewhere [27]. Our study has a number of specific limitations. First, the information we obtained via questionnaires was based on self-reported data, which might induce socially desirable bias and false-positive results. We tried to minimize this by designing factual questions rather than asking for personal opinions as much as possible. By using different data sources, we tried to avoid the common problem of method variance. Second, our aggregation methods could have influenced the outcome of the regression analyses. However, we used validated scales as much as possible. Third, international research on external pressure needs to take account and control for important confounding contextual and local contingent differences across countries that may influence the findings. Therefore, we corrected for country differences in the analyses. So far, we have not been able to find an instrument that aims to include in a single tool the capacity to rank the perceived importance of different types of external pressure. It is not the goal of this paper to validate this instrument, but this publication appears to be the first attempt to measure this concept. Last, we collected quantitative rather than qualitative data. Albeit that qualitative research could give more insight in *what* is discussed when quality performance is on the executive board's agenda, and *how* the required information is acquired and used, for now it is a promising start to have determined the aforementioned relationships quantitatively.

### Practical implications

Executive boards are legally responsible for the hospital's quality performance, and this article demonstrates that they can make a difference. Frequent discussions of quality performance in the executive board room will keep them informed and stimulate the implementation of quality management. Our pan-European study design, including public teaching and non-teaching hospitals, provides important cross-national lessons for hospitals as they seek to improve the quality of care they deliver to patients.

## Conclusions

Having quality on the executive board's agenda allows them to review and discuss quality performance more often in order to improve their hospital's quality management. Generally, and our study supports this, having quality on the executive board's agenda therefore matters.

## Funding

The study, “Deepening our Understanding of Quality Improvement in Europe (DUQuE)” has received funding from the European Community's Seventh Framework Programme (FP7/2007-2013) under grant agreement n° 241822. Funding to pay the Open Access publication charges for this article was provided by European Community's Seventh Framework Programme (FP7/2007-2013) under grant agreement n° 241822.

## Appendix

The DUQuE Project Consortium comprises: N.S.K., D.S. Kringos, M.J.M.H. Lombarts and T. Plochg (Academic Medical Centre—AMC, University of Amsterdam, The Netherlands); M.A. Lopez, M. Secanell, R.S. and P. Vallejo (Avedis Donabedian University Institute-Universitat Autónoma de Barcelona FAD, Red de investigación en servicios de salud en enfermedades crónicas REDISSEC, Spain); P. Bartels and S. Kristensen (Central Denmark Region and Center for Healthcare Improvements, Aalborg University, Denmark); P. Michel and F. Saillour-Glenisson (Comité de la Coordination de l'Evaluation Clinique et de la Qualité en Aquitaine, France); F. Vlcek (Czech Accreditation Committee, Czech Republic); M. Car, S. Jones and E. Klaus (Dr Foster Intelligence—DFI, UK); S. Bottaro and P. Garel (European Hospital and Healthcare Federation-HOPE, Belgium); M. Saluvan (Hacettepe University, Turkey); C. Bruneau and A.D.-L. (Haute Autorité de la Santé-HAS, France); C. Shaw (University of New South Wales, Australia); A. Hammer, O. Ommen and H.P. (Institute of Medical Sociology, Health Services Research and Rehabilitation Science, University of Cologne-IMVR, Germany); O.G. (London School of Hygiene and Tropical Medicine, UK); D.B. and C.W. (The Netherlands Institute for Health Services Research—NIVEL, The Netherlands); H. Kutaj-Wasikowska and B. Kutryba (Polish Society for Quality Promotion in Health Care-TPJ, Poland); A. Escoval and A. Lívio (Portuguese Association for Hospital Development-APDH, Portugal) and M. Eiras, M. Franca and I. Leite (Portuguese Society for Quality in Health Care-SPQS, Portugal); F. Almeman, H. Kus and K. Ozturk (Turkish Society for Quality Improvement in Healthcare-SKID, Turkey); R.M. (University of Birmingham, UK); O.A.A., M.D., C.A. Thompson and A. Wang (University of California, Los Angeles—UCLA, USA); A. Thompson (University of Edinburgh, UK).
